# N-3 Long-Chain Polyunsaturated Fatty Acid Supplementation Significantly Reduces Liver Oxidative Stress in High Fat Induced Steatosis

**DOI:** 10.1371/journal.pone.0046400

**Published:** 2012-10-17

**Authors:** Rodrigo Valenzuela, Alejandra Espinosa, Daniel González-Mañán, Amanda D'Espessailles, Virginia Fernández, Luis A. Videla, Gladys Tapia

**Affiliations:** 1 School of Nutrition and Dietetics, Faculty of Medicine, University of Chile, Santiago, Chile; 2 School of Medical Technology, Faculty of Medicine, University of Chile, Santiago, Chile; 3 Molecular and Clinical Pharmacology Program, Institute of Biomedical Sciences, Faculty of Medicine, University of Chile, Santiago, Chile; Visva Bharati University, India

## Abstract

Omega-3 (*n-3*) long-chain polyunsaturated fatty acids (n-3 LCPUFA) are associated with several physiological functions, suggesting that their administration may prevent non transmissible chronic diseases. Therefore, we investigate whether dietary *n-3* LCPUFA supplementation triggers an antioxidant response preventing liver steatosis in mice fed a high fat diet (HFD) in relation to n-3 LCPUFA levels. Male C57BL/6J mice received (a) control diet (10% fat, 20% protein, 70% carbohydrate), (b) control diet plus *n-3* LCPUFA (108 mg/kg/day eicosapentaenoic acid plus 92 mg/kg/day docosahexaenoic acid), (c) HFD (60% fat, 20% protein, 20% carbohydrate), or (d) HFD plus *n-3* LCPUFA for 12 weeks. Parameters of liver steatosis, glutathione status, protein carbonylation, and fatty acid analysis were determined, concomitantly with insulin resistance and serum tumor necrosis factor-α (TNF-α), interleukin (IL)-1β, and IL-6 levels. HFD significantly increased total fat and triacylglyceride contents with macrovesicular steatosis, concomitantly with higher fasting serum glucose and insulin levels, HOMA, and serum TNF-α, IL-1β, and IL-6. Reduced and total liver glutathione contents were diminished by HFD, with higher GSSG/GSH ratio and protein carbonylation, *n-3* LCPUFA depletion and elevated *n-6*/*n-3* ratio over control values. These changes were either reduced or normalized to control values in animals subjected to HFD and *n-3* LCPUFA, with significant increased hepatic total *n-3* LCPUFA content and reduced *n-6*/*n-3* ratio being observed after *n-3* LCPUFA supplementation alone. So, repletion of liver *n-3* LCPUFA levels by *n-3* LCPUFA dietary supplementation in HFD obese mice reduces hepatic lipid content, with concomitant antioxidant and anti-inflammatory responses favouring insulin sensitivity.

## Introduction

Non-alcoholic fatty liver disease (NAFLD) is characterized by pathological accumulation of fat in the liver in the absence of any other disease related to liver steatosis, which includes a wide spectrum of liver disease ranging from mild asymptomatic fatty liver to non-alcoholic steatohepatitis (NASH) and cirrhosis [1]. NAFLD is considered the hepatic expression of the metabolic syndrome, a condition associated with hypertension, insulin resistance, obesity, and dyslipidemia [2]. Although the pathogenic mechanisms involved in hepatic lipid accumulation are not completely understood, liver steatosis may result from an imbalance between lipid availability, either from enhanced blood uptake and/or de novo lipogenesis, and lipid disposal, either from decreased mitochondrial and peroxisomal fatty acid (FA) β-oxidation and/or reduced ability of lipid output by the liver [3]. The establishment of steatosis in the liver may eventually lead to lipid peroxidation with production of concomitant hepatic injury [4].

NAFLD is characterised by impairment in the bioavailability of liver *n-6* and *n-3* long-chain polyunsaturated fatty acids (LCPUFAs) [5], [6]. Under physiological conditions, the liver is able to synthesize most of LCPUFAs from dietary precursors, which constitute crucial components for both membrane functions, due to their role in establishing adequate membrane fluidity, and signaling functions, acting as second messengers regulating signal transduction processes, with a minor contribution to the energy reserves within the cell [7]. Alterations in liver LCPUFA status in NAFLD are characterised by significant depletion of *n-3* LCPUFA content and enhancement in the *n-6*/*n-3* LCPUFA ratio [5], [6]. These changes also occur in cardiovascular disease [8], obesity [9], and diabetes type II [10], in association with the inflammatory response component of these pathologies [11]. A major factor associated with liver *n-3* LCPUFA depletion in obesity is the development of prolonged oxidative stress, which may be compounded by defective desaturation activity and dietary imbalance, promoting hepatic steatosis [4]–[6].

*N-3* LCPUFAs eicosapentaenoic acid (EPA) and docosahexaenoic acid (DHA) have been associated with key roles in several physiological functions, suggesting that their administration may prevent several non transmissible chronic diseases [12]. In fact, prevention of ischemia/reperfusion liver injury by n-3 LCPUFAs has been established [13]. Furthermore, recent studies suggest that the anti-steatotic effects of EPA and DHA in the liver include directing fatty acids away from triglyceride storage with promotion of their oxidation, as well as an enhanced glucose flux to glycogen synthesis [14]. In the view of these considerations, the present study was aimed to test the hypothesis that dietary *n-3* LCPUFA supplementation triggers antioxidant and anti-inflammatory responses that prevent liver steatosis induced by high fat diet (HFD) administration in mice. For this purpose, parameters related to liver morphological characteristics and levels of serum transaminases, the metabolic syndrome (serum glucose, insulin, HOMA index of insulin resistance, cholesterol, and triglyceride levels), oxidative stress (glutathione status and protein carbonylation), inflammation [serum levels of tumor necrosis factor (TNF)- α, interleukin (IL)-1β, and IL-6] were determined, in relation to liver total fat content and fatty acid composition.

## Materials and Methods

### Ethics Statement

Experimental animal protocols and animal procedures complied with the Guide for the Care and Use of Laboratory Animals (National Academy of Sciences, NIH Publication 6–23, revised 1985) and were approved by the Bioethics Committee for Research in Animals, Faculty of Medicine, University of Chile (CBA 0386 FMUCH).

### Animal preparation and supplementation with n-3 LCPUFA (EPA plus DHA)

Weaning male C57BL/6J mice weighing 12–14 g (Bioterio Central, ICBM, Faculty of Medicine, University of Chile) were randomly assigned to each experimental group and allowed free access to specially formulated control or high fat diets. The composition of the control diet (expressed as % total calories) was 10% fat, 20% protein, and 70% carbohydrate, with a caloric value of 3.85 kcal/g, free of EPA and DHA, and contained 0.7 g of α-linolenic acid (ALA)/100 g of diet. The composition of the HFD was 60% fat, 20% protein, and 20% carbohydrate, with a caloric value of 5.24 kcal/g, free of EPA and DHA, and contained 0.7 g of ALA/100 g of diet (Research Diet INC, Rodent Diet, Product data D12450B and D12492, USA). Animals received water *ad libitum* and were housed on a 12-hour light/dark cycle. From days 1 to 84 (12 weeks), the *n-3* LCPUFA supplemented groups received fish oil (encapsulated fish oil containing 379 mg [EPA+DHA]/g; Acolest TG Product. Procaps, Colombia) through oral administration and the control groups isovolumetric amounts of saline, thus comprising four experimental groups: (a) control diet (control), (b) control diet plus *n-3* LCPUFA, (c) HFD, and (d) HFD plus *n-3* LCPUFA. Under these conditions the *n-3* LCPUFA groups received daily doses of 108 mg/kg of EPA and 92 mg/kg of DHA. Weekly controls of body weight and diet intake were performed through the whole period. At the end of the 12^th^ week, animals were fasted (6–8 h), anesthetized with ketamine and xylazine (150 and 10 mg/kg, respectively), and blood samples were obtained by cardiac puncture for serum AST, ALT, glucose, insulin, IL-1β, IL-6, and TNF-α assessments. Liver samples were frozen in liquid nitrogen (for determination of fatty acid composition) or fixed in phosphate-buffered formalin, embedded in paraffin, and stained with hematoxylin-eosin (for morphology assessment) and analyse by optical microscopy in a blind fashion describing the presence of steatosis and inflammation, both graded as absent, mild, moderated and severe [15].

### Measurements of serum glucose, insulin, cholesterol, triacylglycerol, transaminases (AST and ALT), IL-1β, IL-6 and TNF-α levels

Serum glucose (mM), cholesterol (mg/100 mL), and triacylglycerol levels (g/L) were measured using specific diagnostic kits (Wiener Lab, Argentina). A commercial immunoassay kit for mice serum insulin assessment (µU/mL) was used, according to the manufacturer's instructions (Mercodia, Uppsala, Sweden). Insulin resistance was estimated by the homeostasis model assessment method (HOMA) [fasting insulin (µU/mL)×fasting glucose (mM)/22.5] [16]. Serum aspartate transaminase (AST) and alanine transaminase (ALT) activities (units/L) were measured using specific diagnostic kits (Biomerieux SA, Marcy l^,^Etoile, France). ELISA kits were used for assessment of serum levels (pg/mL) of IL-6, IL-1β and TNF-α (Thermo, Meridian, Rd, USA).

### Liver parameters related to oxidative stress

In anesthetized animals, livers were perfused *in situ* with a cold solution containing 150 mM KCl and 5 mM Tris (pH 7.4) to remove blood for glutathione and protein carbonylation assessments. Reduced glutathione (GSH) and glutathione disulfide (GSSG) contents were assessed with an enzymatic recycling method [17] and protein carbonyl, and total protein contents were spectrophotometrically measured [18].

### Liver total fat and triacylglycerol content and fatty acid analysis

Total lipids were extracted from whole-liver homogenates using a modified Bligh and Dyer extraction procedure [19], and hepatic triacylglycerol content (mg/g liver) was determined using diagnostic kits (Wiener Lab, Argentina). For fatty acid analysis liver samples were homogenized in distilled water and the lipid components were extracted with a 1∶2 chloroform∶methanol solution, followed by centrifugation (2,000×g for 10 minutes at room temperature). After extraction of the chloroformic phase, the solvent was allowed to evaporate and the samples were stored at −20°C [19]. Previous to the gas-liquid chromatography assay, fatty acids from liver phospholipids were methylated by incubation (100°C) with BF_3_ methanol (14%) and the fatty acid methyl esters (FAME) were extracted with hexane. After evaporation with nitrogen and resuspension in dichloromethane, the samples were stored at −20°C until the gas-liquid chromatography assay [20]. A Hewlett Packard gas chromatograph (model 7890A), equipped with a capillary column (J and W DB-FFAP, 30 m×0.25 mm; I.D. 0.25 µm), automatic injector and flame ionization detector, was used for FAME separation and detection. Identification of FAME was carried out by comparison of their retention times with those of individual purified standards, and values were expressed as g/100 g FAME.

### Statistical Analyses

Values shown represent the mean ± SEM for the number of separate experiments indicated. Student^,^s *t*-test for unpaired data or one-way ANOVA and the Newman-Keuls test assessed the statistical significance of differences between mean values. A *P*<0.05 was considered significant.

## Results

### N-3 LCPUFA supplementation reduces HFD-induced increases in body weight, liver weight, and hepatic total fat and triacylglycerol content

Mice subjected to the indicated dietary protocols showed no significant changes in food intake, either at the beginning [assessed at day 2: control diet, 2.1±0.2 g/day (n = 14); control diet+n-3 LCPUFA, 1.8±0.4 (n = 14); HFD, 2.2±0.7 (n = 14); HFD+n-3 LCPUFA, 1.9±0.4 (n = 14)] or at the end of the experimental period [assessed at day 84: control diet, 5.2±0.3 g/day (n = 14); control diet+n-3 LCPUFA, 5.1±0.2 (n = 14); HFD, 5.0±0.4 (n = 14); HFD+n-3 LCPUFA, 4.9±0.4 (n = 14)]. Energy intake in animals subjected to either control diet or HFD were comparable at the beginning [a) control diet, 8.1±1.1 kcal/day (n = 14) versus b) control diet+n-3 LCPUFA, 6.9±1.7 (n = 14); not significant (NS), and c) HFD, 11.5±1.5 (n = 14) versus d) HFD+n-3 LCPUFA, 10.0±2.4 (n = 14); NS] or at the end of treatment [a) control diet, 20.0±1.8 kcal/day (n = 14) versus b) control diet+n-3 LCPUFA, 19.6±1.1 (n = 14); NS, and c) HFD, 26.2±2.3 (n = 14) versus d) HFD+n-3 LCPUFA, 25.7±1.8 (n = 14); NS]. However, energy intake in HFD groups were significantly higher than those in control diet groups [c and d versus a and b; *P*<0.05], independently of n-3 LCPUFA supplementation.

At the end of the 12^th^ week, body weight in HFD was significantly higher (*P*<0.05) in relation to controls (19.8%), control diet plus *n-3* PUFA (36.3%) and HFD plus *n-3* LCPUFA supplemented animals (18.8%) (Table 1). Net increments in body weight in mice subjected to HFD plus *n-3* PUFA (group d) relative to control diet plus *n-3* LCPUFA (group b) [d-b, 3.2±0.93 g (n = 14)] were 41% lower (*P*<0.05) than those in animals receiving HFD diet (group c) relative to controls (group a) [c-a, 5.4±0.3 (n = 14)] (Table 1). Furthermore, HFD elicited a significant enhancement (*P*<0.05) in liver weight over control diet (28%) or control diet plus *n-3* LCPUFA (46%), which was not observed in animals subjected to HFD plus *n-3* LCPUFA (Table 1). Under these conditions, HFD induced significant increments (*P*<0.05) in total liver fat (148%) and triacylglycerol (169%) contents over control values, whereas enhancements of 39% and 49% (*P*<0.05) were observed in animals given HFD plus *n-3* LCPUFA, respectively (Table 1).

**Table 1:** General and biochemical parameters of the experimental groups.

	Groups			
	a) Control diet	b) Control diet+n-3 LCPUFA	c) HFD	d) HFD+n-3 LCPUFA
Liver parameters				
Initial weight (g)	12.8±0.3	12.4±0.5	14.0±0.5	13.5±0.4
Final weight (g)	33.2±0.3^c^	29.2±0.9^c^	39.8±2.2^a,b,d^	33.5±2.1^c^
Liver weight (g)	1.38±0.05^c^	1.20±0.02^c^	1.76±0.05^a,b,d^	1.20±0.08^c^
Total fat (mg/g liver)	39.8±1.8^c,d^	38.4±2.4^c,d^	98.9±4.6^a,b,d^	55.2±3.6^a,b,c^
Triacylglycerol (mg/g liver)	32.7±0.5^c,d^	30.2±0.7^c,d^	87.8±2.9^a,b,d^	48.7±0.3^a,b,c^
Serum parameters				
Fasting glucose (mg/dL)	130±4.8^c^	128±7.9^c^	220±7.1^a,b,d^	126±8.5^c^
Fasting insulin (unit/mL)	5.57±1.6^c^	5.3±1.4^c^	16.6±0.7^a,b,d^	8.3±1.9^c^
HOMA	1.79±0.07^d^	1.68±0.1^d^	9.1±0.51^a,b,d^	2.75±0.16^a,b,c^
Total Cholesterol (mg/dL)	73±3.0^c,d^	60±4.0^c,d^	124±3^a,b^	122±4.0^a,b^
Triacylglycerol (mg/dL)	120±8.0^c^	114±2.0^c,d^	156±4^b,d^	139±2.0^b,c^
AST (U/L)	187.2±11.2	166.0±4.3	189.9±6.8	181.9±5.5
ALT (U/L)	68.1±2.2	71.8±3.6	84.1±2.0	66.1±3.8
IL-6	42.8±8.1^c^	37.6±5.6^c^	67.4±13.0^a,b,d^	48.7±12.8^c^
IL-1β	8.0±3.4^c^	16.1±4.0^c^	42.8±5.9^a,b,d^	21.5±3.9^c^
TNF-α (pg/mL)	26.2±1.0^c^	26.0±1.0^c^	30.3±1.1^a,b,d^	26.6±0.9^c^

Body weight, liver weight, and biochemical variables in control and high fat diet (HFD) fed mice subjected to n-3 long-chain polyunsaturated fatty acid (n-3 LCPUFA) supplementation.

Values represent means ± SEM for 14 mice per experimental group. Significant differences between the groups are indicated by the letters identifying each group (*P*<0.05; one-way ANOVA and the Newman-Keuls' test).

### N-3 LCPUFA supplementation normalized HFD-induced enhancements in serum levels of glucose, insulin, and HOMA values

Mice subjected to HFD exhibited 70% and 200% increases in serum levels of glucose and insulin, respectively, with a consequent 5.1-fold enhancement in HOMA index (*P*<0.05), when compared to control values, effects that were abolished in animals receiving HFD plus *n-3* LCPUFA supplementation (Table 1). Under these conditions, total serum cholesterol was 70% and 67% higher in the HFD and HFD plus *n-3* LCPUFA groups, respectively, as compared to values in controls. However no significant reduction was achieved by *n-3* LCPUFA supplementation in animals fed either control or high fat diets. Interestingly, in animals subjected to HFD *n-3* LCPUFA significantly reduced serum triacylglycerol levels by 11% (Table 1).

### N-3 LCPUFA supplementation suppressed HFD-induced enhancement in serum IL-6, IL-1β, and TNF-α levels, liver steatosis, and liver morphological alterations

**Figure 1:**
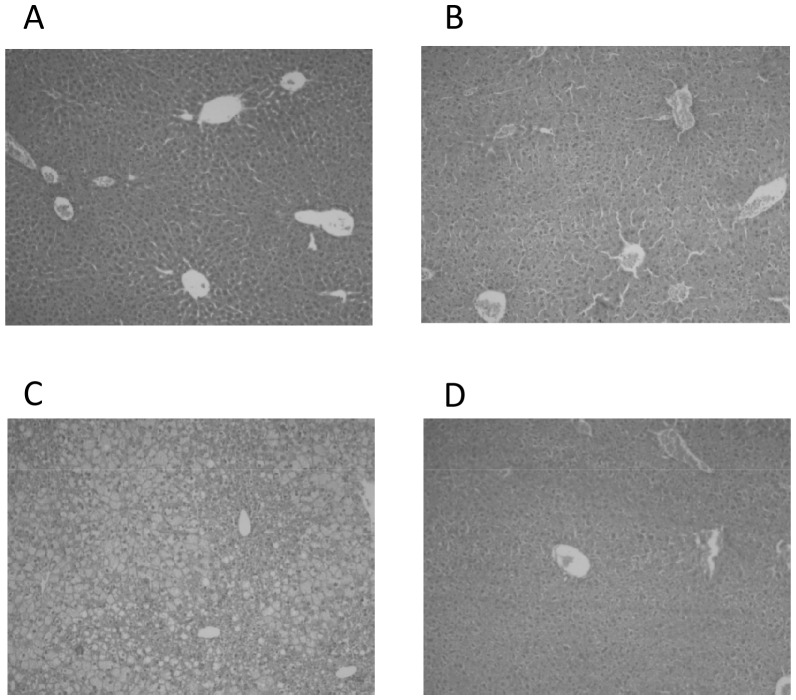
Effect of *n-3* long-chain polyunsaturated fatty acid (*n-3* LCPUFA) supplementation on liver histology. Representative liver sections from animals given (A) control diet, (B) control diet plus *n-3* LCPUFA, (C) HFD, and (D) HFD plus *n-3* LCPUFA (hematoxylin-eosin liver sections from a total of 9 animals per experimental group; original magnification ×40).

Experimental groups exhibited no significant differences in serum AST and ALT activities. In relation to controls, the HFD group exhibited significantly enhanced (*P*<0.05) serum levels of IL-6, IL-1β, and TNF-α (57%, 436%, and 16%, respectively), an effect that was suppressed by *n-3* LCPUFA supplementation in the HFD plus *n-3* LCPUFA group (Table 1). Mice subjected to control diet (Figure 1A) and control diet plus *n-3* LCPUFA (Figure 1B) exhibited normal histology, whereas those in the HFD group showed macro and microvesicular steatosis, arquitectural distortion with moderate periportal and lobular inflammation and necrosis foci (Figure 1C). *N-3* LCPUFA supplementation reverted hepatic steatosis, with persistence of only few steatosis foci, absence of inflammation, and modest arquitectural distortion (Figure 1D).

### HFD-induced changes in liver oxidative stress-related parameters are abolished by n-3 LCPUFA supplementation

**Figure 2:**
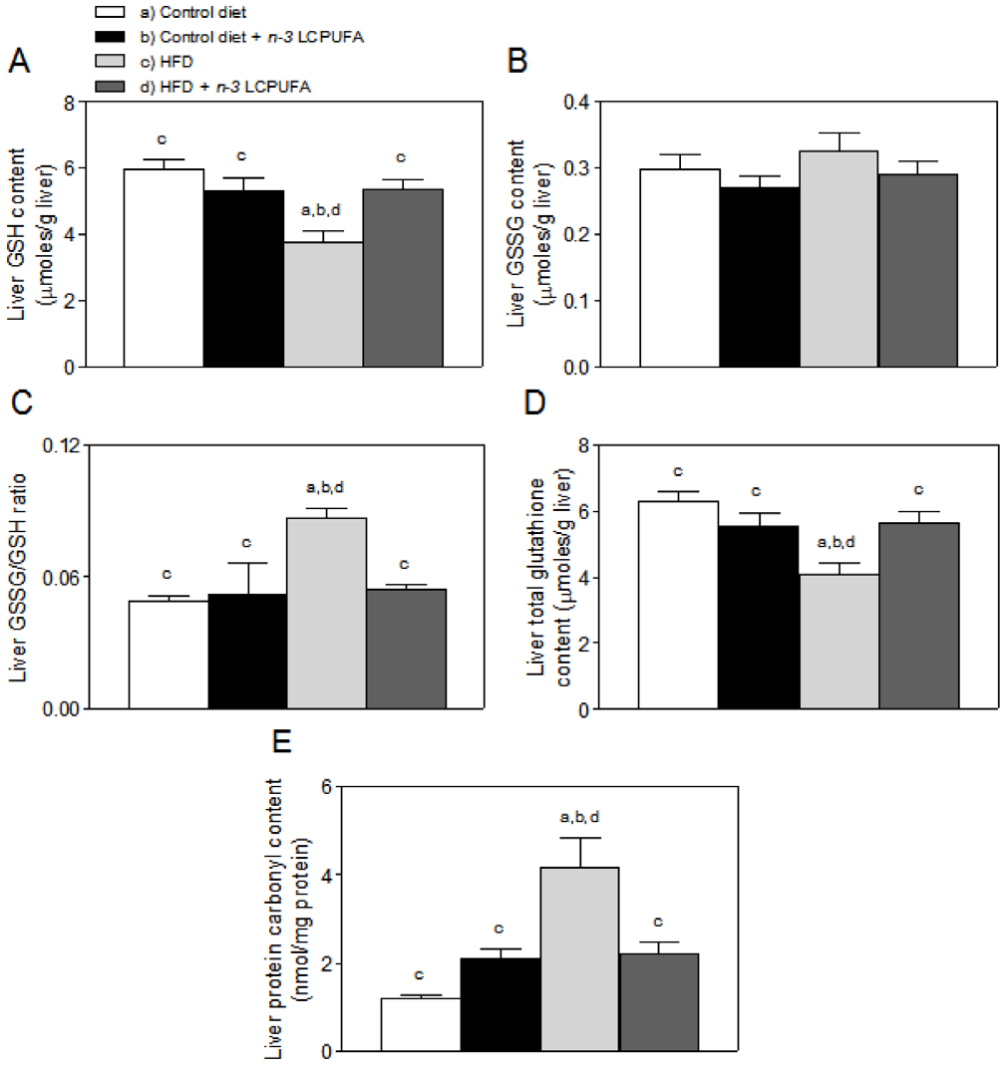
Effect of *n-3* long-chain polyunsaturated fatty acid (*n-3* LCPUFA) supplementation in oxidative stress parameters. (A) Reduced glutathione (GSH), (B) oxidized glutathione (GSSG), (C) GSSG/GSH ratio, (D) total glutathione (GSH+2GSSG), (E) and protein carbonyls. Values shown represent means ± SEM for six mice per experimental group. Significant differences between the groups are indicated by the letters identifying each group (*P*<0.05; ANOVA and the Newman-Keuls' test).

Animals subjected to control diet and control diet plus *n-3* LCPUFA administration exhibited comparable values in liver GSH content (Figure 2A), GSSG levels (Figure 2B), GSSG/GSH ratio (Figure 2C), total glutathione content (Figure 2D), and protein carbonyl levels (Figure 2E). HFD induced significant 36% diminution in hepatic GSH (Figure 2A) and total glutathione content (Figure 2D), with 78% and 245% increases in GSSG/GSH ratio (Figure 2C) and protein carbonyl content (Figure 2E), respectively, without altering liver GSSG levels (Figure 2B), changes that were abolished by combined HFD plus *n-3* LCPUFA supplementation.

### Effects of HFD and n-3 LCPUFA supplementation on liver fatty acid composition

**Figure 3:**
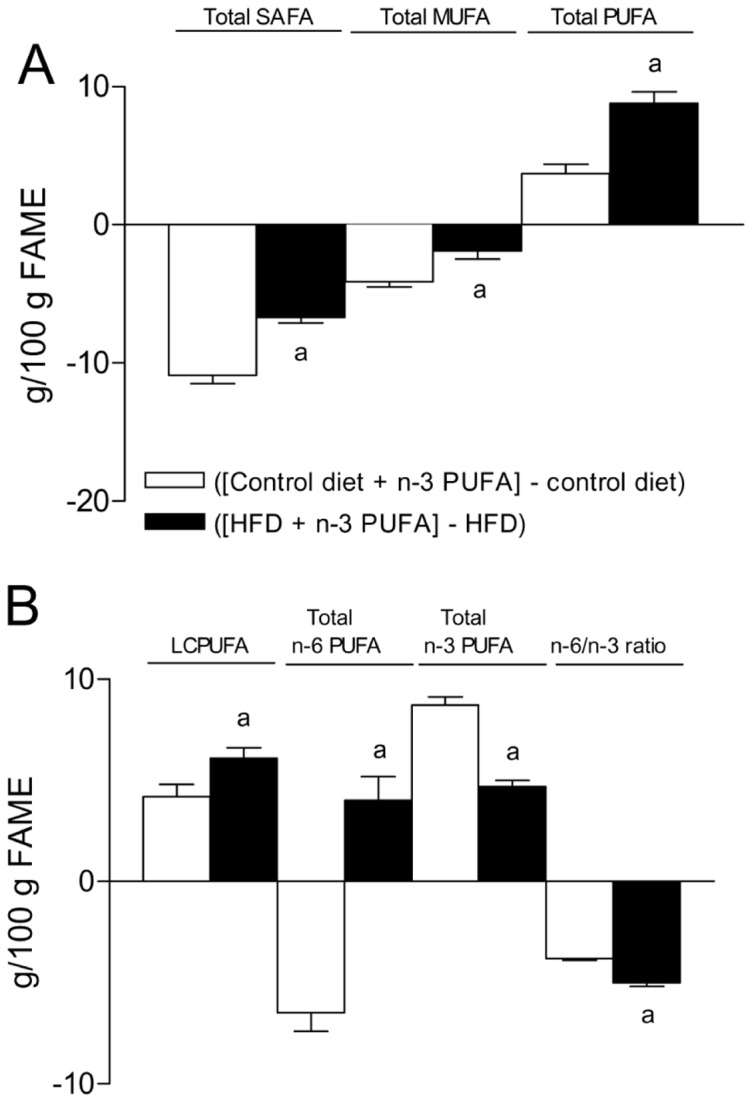
Net changes in liver fatty acids. (A) Total saturated fatty acids (SAFA), monounsaturated FA (MUFA), polyunsaturated FA (PUFA) and (B) long-chain polyunsaturated FA (LCPUFA), total n-6 PUFA, total n-3 PUFA, and n-6/n-3 ratio induced by high fat diet (HFD) in non-supplemented mice and animals subjected to n-3 LCPUFA supplementation. Data was calculated by subtracting the mean values in the control diet group from individual values in the group subjected to control diet supplemented with n-3 LCPUFA and the mean values in the HFD group from individual values in the group given HFD and n-3 LCPUFA. Values shown represent means ± SEM for nine mice per experimental group. ^a^ *P*<0.05 assessed by Student^,^s *t*-test for unpaired data.

N-3 LCPUFA supplementation in mice subjected to control diet led to 28% decrease (*P*<0.05) in the content of total SAFA of the liver, due to significant reductions in 14∶0, 16∶0, and 18∶0 compare to control diet, without changes in that of total MUFA and total PUFA, whereas LCPUFA and total *n-3* PUFA were enhanced (*P*<0.05) (Table 2). Under these conditions, the total content of *n-6* PUFA decreased by 23% (*P*<0.05), with a net 70% diminution in the *n-6*/*n-3* PUFA ratio (Table 2). Mice supplemented with HFD showed 17% enhancement (*P*<0.05) in liver total SAFA content over animals given control diet, without changes in that of total MUFA, whereas total PUFA, LCPUFA, total *n-6* PUFA, and total *n-3* PUFA were reduced by 31%, 38%, 31%, and 56%, respectively (Table 2). Under these conditions, the hepatic *n-6*/*n-3* PUFA ratio was enhanced by 57% (*P*<0.05) (Table 2). Combined HFD and *n-3* LCPUFA supplementation elicited comparable levels of total hepatic SAFA, MUFA, PUFA, LCPUFA, *n-6* PUFA, and *n-3* PUFA to those elicited by control diet, concomitantly with 36% decrease (*P*<0.05) in the *n-6*/*n-3* PUFA ratio of the liver (Table 2). However, when the net differences in fatty acid liver composition are considered [(control diet+*n-3* LCPUFA)−control diet] versus (HFD+*n-3* LCPUFA)−HFD], *n-3* PUFA supplementation elicited 39% and 54% reduction in total SAFA and MUFA (Figure 3A), respectively, with 138% (Figure 3A) and 45% (Figure 3B) increases in total PUFA and in LCPUFA, respectively (*P*<0.05). In addition, total *n-3* PUFA decreased by 46%, whereas decrease in the *n-6*/*n-3* ratio was further elevated by 32% (*P*<0.05; Figure 3B).

**Table 2:** Fatty acid composition of liver total lipids.

	Fatty acid composition (g/100g FAME)			
	Groups			
Fatty acid	a) Control diet	b) Control diet+n-3 LCPUFA	c) HFD	d) HFD+n-3 LCPUFA
14∶0	2.18±0.3^b,d^	1.33±0.2^a,c^	2.19±0.2^b,d^	1.36±0.16^a,c^
14∶1, n-7	0.40±0.03^c^	0.36±0.1^c^	1.71±0.1^a,b,d^	0.37±0.04^c^
16∶0	22.7±0.07^b^	15.4±2.0^a,c^	25.3±0.5^b^	22.7±0.4^b^
16∶1, n-7	2.30±0.14^b,c,d^	1.40±0.2^a,c^	5.23±0.2^a,b,d^	3.61±0.1^a,b,c^
18∶0	12.7±0.3^b^	10.4±1.3^a,c,d^	14.6±0.6^b^	13.5±0.2^b^
18∶1, n-9	22.5±0.6	20.4±2.6	23.3±0.5	23.6±0.5
18∶2, n-6	12.6±0.5	11.8±1.5	9.72±0.6	11.8±0.5
18∶3, n-3	0.30±0.02	0.35±0.1	0.24±0.03	0.33±0.1
20∶4, n-6	11.9±0.6^b,c,d^	7.71±1.0^a^	8.77±0.3^a^	8.1±0.4^a^
20∶5, n-3	0.49±0.1^c^	5.21±0.02^a,c,d^	0.21±0.03^b,d^	0.97±0.2^a,b,c^
22∶5, n-3	0.70±0.14^c^	1.18±0.2^a,c,d^	0.26±0.1^a,b,d^	0.84±0.1^b,c^
22∶6, n-3	3.90±0.2^c^	7.76±1.0^a,c,d^	1.65±0.1^a,b,d^	5.04±0.2^b,c^
Total SAFA	38.7±0.9^b,c^	27.8±3.5^a,c^	45.2±1.2^a,b^	38.5±0.4^b^
Total MUFA	27.1±0.6	23.0±2.9^c,d^	31.0±0.6^b^	29.1±0.6^b^
Total PUFA	34.2±0.9^c^	37.9±4.8^c^	23.7±0.8^a,b,d^	32.5±0.8^c^
LCPUFA	20.5±0.9^c,b^	24.7±3.1^a,c,d^	12.8±0.3^a,b,d^	18.9±0.5^c,b^
Total n-6 PUFA	28.7±1.0^b,c^	22.2±2.9^a^	19.9±1.5^a^	23.9±1.1
Total n-3 PUFA	5.48±0.2^c^	14.2±1.^a,c,d^	2.41±0.2^a,b,d^	7.11±0.3^c,b^
n-6/n-3 ratio	5.35±0.3^b,d^	1.58±0.1^a,c,d^	8.38±0.6^a,b,d^	3.42±0.2^a,b,c^

Fatty acid composition of liver total lipids in control and high fat diet (HFD) fed mice subjected to n-3 long-chain polyunsaturated fatty acid (n-3 LCPUFA) supplementation. Values represent means ± SEM for nine mice per experimental group. Significant differences between the groups are indicated by the letters identifying each group (*P*<0.05; ANOVA and the Newman-Keuls' test). Saturated fatty acid (SAFA) ) are 12∶0, 14∶0, 16∶0 and 18∶0. Mono-unsaturated fatty acids (MUFA) are 14∶1,n-7, 16∶1,n-7 and 18∶1.Poly-unsaturated fatty acids (PUFA) are 18∶2,n-6 and 18∶3,n-3. Long-chain poly-unsaturated fatty acids are 20∶4,n-6, 20∶5n-3 (eicosapentaenoic acid, EPA), 22∶5,n-3, and 22∶6,n-3 (docosahexaenoic acid, DHA).

## Discussion

N-3 supplementation in mice subjected to HFD for 12 weeks led to significant reduction in body weight compared to unsupplemented animals, probably associated with lower adiposity due to PPAR-α activation favouring fatty acid oxidation [21]. HFD-induced macro and microvesicular hepatic steatosis due to substantial enhancement in total fat and triacylglycerol contents of the liver. These alterations are associated with the onset of oxidative stress in the liver, as evidenced by (i) significant changes in the glutathione status, namely, reduction in GSH content with enhancement in the GSSG/GSH ratio; and (ii) a 3.5-fold increase in protein carbonylation over control values. Liver GSSG levels in HFD-treated rats were comparable to controls, a finding that may be explained in terms of sinusoidal and/or canalicular efflux [22], in agreement with the net diminution in total glutathione content induced by HFD. HFD-induced liver oxidative stress is associated with progressively increasing availability and oxidation of FAs in the liver [4] and/or TNF-α-induced enhancement in mitochondrial reactive oxygen species (ROS) production [23], [24], which is related to liver n-3 PUFA and n-6 PUFA depletion with enhanced n-6/n-3 ratio, and insulin resistance with hyperinsulinemic response [9], [25]. Under these conditions. HFD-induced depletion of hepatic n-3 and n-6 PUFA is associated with consumption due to ROS-dependent lipid peroxidation and/or decreased *de novo* biosynthesis due to down-regulation of Δ6 and Δ5 desaturases triggered by insulin resistance [4], [6]. Underlying steatotic mechanisms induced by HFD include (i) insulin resistance-dependent peripheral lipolysis and FA mobilization to the liver [26], [27]; and (ii) stimulation of hepatic *de novo* synthesis of saturated FAs under conditions of liver n-3 LCPUFA depletion [28] and hyperinsulinemic response [29].

Dietary *n-3* LCPUFA supplementation [200 mg/kg/day) in mice subjected to HFD substantially prevents the accumulation of fat in the liver, with concomitant normalization of insulin resistance, in agreement with studies in *ob/ob* mice [30]. Several mechanisms may be involved in the anti-steatotic action of *n-3* LCPUFA supplementation. Acting as signaling molecules regulating hepatic lipid metabolism, *n-3* LCPUFA down-regulate the expression of transcription factor sterol regulatory element-binding protein 1c (SREBP-1c) and its processing, with inhibition of the transcription of lipogenic genes (fatty acid synthase, acetyl-CoA carboxylase, stearoyl-CoA desaturase-1), and consequent reduction in *de novo* lipogenesis, whereas activation of peroxisome proliferator-activated receptor-α (PPAR-α) triggers FA oxidation [7]. In addition, *n-3* LCPUFA may enhance the antioxidant potential of the liver, acting through direct and/or indirect mechanisms, a condition proposed to improve insulin sensitivity [31]. Considering that *n-3* LCPUFA are highly susceptible to free-radical reactions [4], [13], a direct antioxidant action is evidenced by the net decrease of 4.02 g *n-3* LCPUFA/100 g FAME induced by HFD in the liver of *n-3* LCPUFA supplemented mice over those in non-supplemented animals (from Table 1), an effect that is not observed for *n-6* PUFA. Indirect antioxidant action of *n-3* LCPUFA supplementation may involve up-regulation of the expression of antioxidant proteins such as heme-oxygenase, glutamate cysteine ligase [32], glutathione peroxidase, glutathione reductase, glutathione-S-transferase, and catalase [33], a mechanism associated with activation of NF-E2-related factor 2 (Nrf2) by *n-3* LCPUFA oxidation products [32]. In agreement with these views, *n-3* LCPUFA-induced antioxidant potential is associated with normalization of insulin levels, insulin sensitivity, and glucose homeostasis altered by HFD. Besides the significant reduction in hepatic steatosis, mice subjected to HFD supplemented with *n-3* LCPUFA exhibited a diminution in weight gain compared to those given HFD alone. Weight loss was shown to improve liver and erythrocyte *n-3* LCPUFA status of obese patients, with improvement in biomarkers of oxidative stress, membrane FA insaturation, and *n-3* LCPUFA biosynthetic capacity, thus representing a crucial therapeutic issue in the improvement of obesity-related metabolic alterations [34]. In agreement with this contention, *n-3* LCPUFA supplementation reduces hepatic steatosis in obese patients, as evidenced by ultrasonography [14], [35]–[37] or direct assessment in post-treatment liver biopsies [38].

Mice subjected to HFD showed enhanced pro-inflammatory cytokine signaling evidenced by significant increases in serum TNF-α, IL-1β, and IL-6 levels, with development of moderate inflammation, responses that were suppressed by n-3 LCPUFA supplementation. Studies in NAFLD patients revealed that *n-3* LCPUFA administration decreased serum transaminases [35], [38], TNF-α [35], soluble TNF receptor 1 and 2 levels, and oxidative stress markers [38], with improvement in hepatic steatosis [35], [38], fibrosis, hepatocyte ballooning, and lobular inflammation in 85% of the patients [38]. These observations point to an anti-inflammatory effect of *n-3* LCPUFA supplementation, which may involve down-regulation of inflammatory gene expression. The latter feature may be due to *n-3* LCPUFA activation of PPAR-α, followed by PPAR-α-mediated inactivation of pro-inflammatory nuclear factor-κB (NF-κB) and/or activating protein 1 (AP-1) through inactive complex formation with either NF-κB p65 or AP-1 c-Jun [39]. The anti-inflammatory effects of *n-3* LCPUFA may be also mediated by resolvins and protectins, derived either from EPA (E series) or DHA (D series) [40], [41], as shown for resolving D1 and protectin D1 enhanced formation in adipose tissue from n-3 LCPUFA supplemented *ob/ob* mice [42] over control values. Alternatively, targeting the G-protein-coupled receptor GPR120 by *n-3* LCPUFA leads to potent anti-inflammatory and insulin-sensitizing effects in macrophages and adipocytes [43]. This signaling pathway of *n-3* LCPUFA involves recruitment of β-arrestin 2 to membrane bound GPR120 and internalization of the GPR120-β-arrestin 2 complex, which upon interaction with transforming growth factor β (TGF-β) activated kinase 1 binding protein 1 (TAB1) inhibits the TAB1 interaction with TGF-β activated kinase 1 (TAK1), thus abrogating inflammation [44]. Effects of n-3 LCPUFA supplementation on NASH features are currently being addressed by randomized, controlled trials [42].

Collectively, data reported support a role for n-3 LCPUFA (EPA+DHA) dietary supplementation in reducing hepatic lipid content in mice subjected to HFD, which may underlie (i) direct and indirect antioxidant responses and *n-3* LCPUFA repletion favoring insulin sensitivity; (ii) PPAR-α activation and SREBP-1c down-regulation favoring FA oxidation over lipogenesis; and (iii) an anti-inflammatory response mediated by either *n-3* LCPUFA-derived resolvins and protectins and/or direct PPAR-α-dependent NF-κB and AP-1 inhibition. Data presented strongly support the potential therapeutic use of *n-3* LCPUFA supplementation in the treatment of human liver steatosis induced by nutritional factors or other etiologies.

## References

[1] Bellentani S, Marino M (2009) Epidemiology and natural history of non-alcoholic fatty liver disease (NAFLD). Ann Hepatol 8: S4–S8. 19381118

[2] Alberti KG, Zimmet P, Shaw J (2005) IDF Epidemiology Task Force Consensus Group (2005) The metabolic syndrome - a new worldwide definition. Lancet 366: 1059–1062. 16182882 10.1016/S0140-6736(05)67402-8

[3] Musso G, Gambino R, Cassader M (2009) Recent insights into hepatic lipid metabolism in non-alcoholic fatty liver disease (NAFLD). Progr Lipid Res 48: 1–26. 10.1016/j.plipres.2008.08.001 18824034

[4] Videla LA, Rodrigo R, Araya J, Poniachik J (2006) Insulin resistance and oxidative stress interdependency in non-alcoholic fatty liver disease. Trends Mol Med 12: 555–558. 17049925 10.1016/j.molmed.2006.10.001

[5] Araya J, Rodrigo R, Videla LA, Thielemann L, Pettinelli P, et al (2004) Increase in long-chain polyunsaturated fatty acid n-6/n-3 ratio in relation to hepatic steatosis in patients with non-alcoholic fatty liver disease. Clin Sci 106: 635–643. 14720121 10.1042/CS20030326

[6] Araya J, Rodrigo R, Pettinelli P, Araya AV, Poniachik J, et al (2010) Decreased liver fatty acid Δ-6 and Δ-5 desaturase activity in obese patients. Obesity 18: 1460–1463. 19875987 10.1038/oby.2009.379

[7] Brenner RR (2003) Hormonal modulation of Δ6 and Δ5 desaturases: case of diabetes. Prostag Leukotr Ess 68: 151–162. 10.1016/s0952-3278(02)00265-x 12538079

[8] Warensjö E, Sundström J, Vessby B, Cederholm T, Risérus U (2008) Markers of dietary fat quality and fatty acid desaturation as predictors of total and cardiovascular mortality: a population-based prospective study. Am J Clin Nutr 88: 203–209. 18614742 10.1093/ajcn/88.1.203

[9] Zhou YE, Kubow S, Dewailly E, Julien P, Egeland GM (2009) Decreased activity of desaturase 5 in association with obesity and insulin resistance aggravates declining long-chain n-3 fatty acid status in Cree undergoing dietary transition. Brit J Nutr 102: 888–894. 19338705 10.1017/S0007114509301609

[10] Delarue J, LeFoll C, Corporeau C, Lucas D (2004) N-3 long-chain polyunsaturated fatty acids: a nutritional tool to prevent insulin resistance associated to type 2 diabetes and obesity? Rep Nutr Develop 44: 289–299. 10.1051/rnd:2004033 15460168

[11] Martinelli N, Girelli D, Malerba G, Guarini P, Illig T, et al (2008) FADS genotypes and desaturase activity estimated by the ratio of arachidonic acid to linolénico acid are associated with inflammation and coronary artery disease. Am J Clin Nutr 88: 941–949. 18842780 10.1093/ajcn/88.4.941

[12] Simopoulos AP (2008) The importance of the omega-6/omega-3 fatty acid ratio in cardiovascular disease and other chronic diseases. Exp Biol Med 233: 674–688. 10.3181/0711-MR-311 18408140

[13] Zúñiga J, Venegas F, Villarreal M, Núñez D, Chandía M, et al (2010) Protection against in vivo liver ischemia-reperfusion injury by n-3 long-chain polyunsaturated fatty acids in the rat. Free Radic Res 44: 854–863. 20528561 10.3109/10715762.2010.485995

[14] Capanni M, Calella F, Biagini MR, Genise S, Raimondi L, et al (2006) Prolonged n-3 polyunsaturated fatty acid supplementation ameliorates hepatic steatosis in patients with non-alcoholic fatty liver disease: a pilot study. Aliment Pharmacol Ther 23: 1143–1151. 16611275 10.1111/j.1365-2036.2006.02885.x

[15] Piro S, Spadaro L, Russello M, Spampinato D, Oliveri CE, et al (2008) Molecular determinants of insulin resistance, cell apoptosis and lipid accumulation in non-alcoholic steatohepatitis. Nutr Metab Cardiovasc Dis 18: 545–552. 18063353 10.1016/j.numecd.2007.08.002

[16] Matthews DR, Hosker JP, Rudenski AS, Naylor BA, Treacher DF, et al (1985) Homeostasis model assessment: insulin resistance and β-cell function from fasting glucose and insulin concentration in man. Diabetologia 28: 412–419. 3899825 10.1007/BF00280883

[17] Rahman I, Kode A, Biswas SK (2006) Assay for quantitative determination of glutathione and glutatione disulfide levels using enzymatic recycling method. Nature Protocols 1: 3159–3165. 17406579 10.1038/nprot.2006.378

[18] Reznick AZ, Packer L (1994) Oxidative damage to proteins: spectrophotometric method for carbonyl assay. Methods Enzymol 233: 357–363. 8015470 10.1016/s0076-6879(94)33041-7

[19] Bligh EG, Dyer WJ (1959) A rapid method of total lipid extraction and purification. Canadian J Biochem Physiol 37: 911–917. 10.1139/o59-099 13671378

[20] Morrison WR, Smith LM (1964) Preparation of fatty acid methyl esters and dimethylacetals from lipids with fluoride-methanol. J Lipid Res 5: 600–608. 14221106

[21] Poudyal H, Panchal SK, Diwan V, Brown L (2011) Omega-3 fatty acids and metabolic syndrome: effects and emerging mechanisms of action. Prog Lipid Res 50: 372–387. 21762726 10.1016/j.plipres.2011.06.003

[22] DeLeve LD, Kaplowitz N (1991) Glutathione metabolism and its role in hepatotoxicity. Pharmacol Ther 52: 287–305. 1820580 10.1016/0163-7258(91)90029-l

[23] Aronis A, Madar Z, Tirosh O (2005) Mechanism underlying oxidative stress-mediated lipotoxicity: exposure of J774.2 macrophages to triacylglycerols facilitates mitocondrial reactive oxygen species production and cellular necrosis. Free Radic Biol Med 38: 1221–1230. 15808420 10.1016/j.freeradbiomed.2005.01.015

[24] Schulze-Osthoff K, Bakker AC, Vanhaesebroeck B, Beyaert R, Jacob WA, et al (1992) Cytotoxic activity of tumor necrosis factor is mediated by early damage to mitochondrial functions. Evidence for the involvement of mitochondrial radical generation. J Biol Chem 267: 5317–5323. 1312087

[25] Allard JP, Aghdassi E, Mohammed S, Raman M, Avand G, et al (2008) Nutritional assessment and hepatic fatty acid composition in non-alcoholic fatty liver disease (NAFLD): a cross-sectional study. J Hepatol 48: 300–307. 18086506 10.1016/j.jhep.2007.09.009

[26] Nielsen S, Guo Z, Johnson CM, Hensrud D, Jensen MD (2004) Splanchnic lipolysis in human obesity. J Clin Invest 113: 1582–1588. 15173884 10.1172/JCI21047 PMC419492

[27] Fabbrini E, Mohammed BS, Magkos F, Korenblat KM, Patterson BW, et al (2008) Alterations in adipose tissue and hepatic lipid kinetics in obese men and women with nonalcoholic fatty liver disease. Gastroenterology 134: 424–431. 18242210 10.1053/j.gastro.2007.11.038 PMC2705923

[28] Pettinelli P, del Pozo T, Araya J, Rodrigo R, Araya AV, et al (2009) Enhancement in liver SREBP-1c/PPAR-α ratio and steatosis in obese patients: correlations with insulin resistance and n-3 long-chain polyunsaturated fatty acid depletion. Biochim Biophys Acta 1792: 1080–1086. 19733654 10.1016/j.bbadis.2009.08.015

[29] Pettinelli P, Videla LA (2011) Up-regulation of PPAR-γ mRNA expression in the liver of obese patients: an additional reinforcing lipogenic mechanism to SREBP-1c induction. J Clin Endocrinol Metab 96: 1424–1430. 21325464 10.1210/jc.2010-2129

[30] González-Périz A, Horrillo R, Ferré N, Gronert K, Dong B, et al (2009) Obesity-induced insulin resistance and hepatic steatosis are alleviated by ω-3 fatty acids: a role for resolvins and protectins. FASEB J 23: 1946–1957. 19211925 10.1096/fj.08-125674 PMC2698663

[31] Houstis N, Rosen ED, Lander ES (2006) Reactive oxygen species have a causal role in multiple forms of insulin resistance. Nature 440: 944–948. 16612386 10.1038/nature04634

[32] Gao L, Wang J, Sekhar KR, Yin H, Yared NF, et al (2007) Novel n-3 fatty acid oxidation products activate Nrf2 by destabilizing the association between Keap1 and Cullin3. J Biol Chem 282: 2529–2537. 17127771 10.1074/jbc.M607622200

[33] Demoz A, Willumsen N, Berge RK (1992) Eicosapentaenoic acid at hypotriglyceridemic dose enhances the hepatic antioxidant defense in mice. Lipids 27: 968–971. 1487958 10.1007/BF02535573

[34] Elizondo A, Araya J, Rodrigo R, Signorini C, Sgherri C, et al (2008) Effects of weight loss on liver and erythrocyte polyunsaturated fatty acid pattern and oxidative stress status in obese patients with non-alcoholic fatty liver disease. Biol Res 41: 59–68. 18769764

[35] Spadaro L, Magliocco O, Spampinato D, Piro S, Oliveri C, et al (2008) Effects of n-3 polyunsaturated fatty acids in subjects with nonalcoholic fatty liver disease. Dig Liver Dis 40: 194–199. 18054848 10.1016/j.dld.2007.10.003

[36] Zhu FS, Liu S, Chen XM, Huang ZG, Zhang DW (2008) Effects of n-3 polyunsaturated fatty acids from seal oils on nonalcoholic fatty liver disease associated with hyperlipemia. World J Gastroenterol 14: 6395–6400. 19009658 10.3748/wjg.14.6395 PMC2766124

[37] Hatzitolios A, Savopoulos C, Lazaraki G, Sidiropoulos I, Haritanti P, et al (2004) Efficacy of omega-3 fatty acids, atorvastatin, and orlistat in non-alcoholic fatty liver disease with dyslipidemia. Indian J Gastroenterol 23: 131–134. 15333967

[38] Tanaka N, Sano K, Horiuchi A, Tanaka E, Kiyosawa K, et al (2008) Highly purified eicosapentaenoic acid treatment improves nonalcoholic steatohepatitis. J Clin Gastroenterol 42: 413–418. 18277895 10.1097/MCG.0b013e31815591aa

[39] Jump DB (2008) N-3 polyunsaturated fatty acid regulation of hepatic gene transcription. Curr Opin Lipidol 19: 242–247. 18460914 10.1097/MOL.0b013e3282ffaf6a PMC2764370

[40] Serhan Ch, Chiang N, Van Dyke T (2008) Resolving inflammation: dual anti-inflammatory and pro-resolution lipid mediators. Nat Rev Inmunol 8: 349–361. 10.1038/nri2294 PMC2744593 18437155

[41] Kalupahana NS, Claycombe K, Newman SJ, Stewart T, Siriwardhana N, et al (2010) Eicosapentaenoic acid prevents and reverses insulin resistance in high-fat diet- induced obese mice via modulation of adipose tissue inflammation. J Nutr 140: 1915–1922. 20861209 10.3945/jn.110.125732

[42] Shapiro H, Tehilla M, Attal-Singer J, Bruck R, Luzzatti R, et al (2011) The therapeutic potential of long-chain omega-3 fatty acids in nonalcoholic fatty liver disease. Clin Nutr 30: 6–19. 20619513 10.1016/j.clnu.2010.06.001

[43] Oh DY, Talukdar S, Bae EJ, Imamura T, Morinaga H, et al (2010) GPR120 is an omega-3 fatty acid receptor mediating potent anti-inflammatory and insulin-sensitizing effects. Cell 142: 687–698. 20813258 10.1016/j.cell.2010.07.041 PMC2956412

[44] Talukdar S, Olefsky JM, Osborn O (2011) Targeting GPR120 and other fatty acid-sensing GPCRs ameliorates insulin resistance and inflammatory diseases. Trends Pharmacol Sci 32: 543–555. 21663979 10.1016/j.tips.2011.04.004 PMC3419590

